# Why does the multidisciplinary study of beliefs and believing matter so much?

**DOI:** 10.3389/fpsyg.2022.946953

**Published:** 2022-07-28

**Authors:** Lluis Oviedo

**Affiliations:** Theology Department, Antonianum University, Rome, Italy

**Keywords:** beliefs, social systems, values, meaning, politics, economy

## Introduction

Considerable progress has been made in recent years in the study of beliefs and the believing process. Several programs are trying to better understand this cognitive function and its undeniable contribution to human development and success. The time has now come to better place the dynamics of believing in connection with other cognitive functions and with social systems. Indeed, we are becoming more aware of the important role that beliefs play as a central dimension in human cognition and behavior, about the function of shared beliefs in the stability of social systems and in human interaction and communication. More research is needed to better describe how beliefs and believing contribute to humans dealing with their own environment and other people; to keep working social systems, like the economy, politics, science, education, the judiciary, and obviously religion; and how such sets of beliefs are connected with those social structures. Believing can be observed as a clear case in which the psychological dimension appears as entrenched with the social, rendering those social systems viable; indeed those beliefs appear paramount for the formation of such social systems.

The present short reflection neglects the issue of the role believing plays in general cognition, an issue that has been intensely researched in cognitive sciences and epistemology. In what follows, the focus will lay on the social dimensions linked to belief and believing.

To clarify the concept of belief, some standard dictionaries provide clear definitions; for instance, the *Merriam-Webster* offers the following: “a state or habit of mind in which trust or confidence is placed in some person or thing”; while the *Oxford English Dictionary* offers this one: “a strong feeling that something/somebody exists or is true; confidence that something/somebody is good or right.” As can be appreciated, different dimensions converge, which include cognition, which points to the true value, confidence or trust, and finally even goodness.

## How important are beliefs for society at large and its sustainability?

A recent article about developments in fundamental physics, published in the weekly news magazine *The Economist*, introduced the topic with the words “By abandoning some long-held beliefs, physicists are clearing a path to the future” (28 August 2021, p. 63). More recently, the same periodical was published in a section dedicated to economic analysis an article with the title “War and wokery,” about how the recent international conditions are pressing for a greater ethical engagement in the economy. The article used four times the words “belief” or “believes,” and one “faith.” The author states, for instance: “Mr. Sonnenfeld […] has become the high priest of a belief system in Western business…” (*The Economist*, 2 April 2022, p. 63). A different case emerges in politics. For example, the recent book of Cass Sunstein, *This is Not Normal: The Politics of Everyday Expectations* (Sunstein, 2021), insists on how much democracy depends on the beliefs of people, beliefs which are quite unstable and changeable, even if in other cases they become more resistant and work as group markers, like the religious ones.

The quoted cases support Agustin Fuentes, who in his recent book *Why We Believe?* (Fuentes, 2019), claimed that science and economics are systems of beliefs too and that this condition invites us to consider both critical spheres of human activity in a different light, or within a specific framework, beyond the certainties and strengths that science and the economy have always claimed. Everything gets a new light and deserves a different treatment when it is assumed that the cognitive model these realities follow falls more on the side of believing than the one of factual and tested knowledge. This step is quite remarkable if we keep in mind that until quite recently, talk or titles about beliefs and believing usually related to religious or supernatural views and deeply held convictions (Shermer, 2011; Shagan, 2021). Indeed, what is surprising in Fuentes’s book is that it is not a book about religion, not only.

We are now becoming aware of the extent of belief and how the believing process informs most aspects of human cognition. Indeed, much progress has been achieved when we consider that believing is not only concerned with religious faith and practice, or with broadly held values and meaning. Rather, believing is concerned with a cognitive dimension that is involved in many aspects of human life and social systems. Such a discovery has been brought about by a much more accurate analysis and understanding of the process of believing and a general acknowledgment of the impact of biases in academic life. Such awareness could render beliefs or believing a less reliable cognitive activity, one all too often troubled by deception and delusions. Since Aristotle, believing has been contrasted with knowing, based on strict epistemic methods; a rather second-class cognition, reserved for other areas where the ideal model was harder to attain (Miller, 2013).

Rescuing the meaning and value of believing has not been easy. The previously quoted book of Agustin Fuentes has given us important insight and nourished a new interest in this field. Ongoing projects, like *Creditions*, based in Graz University aimed at researching the belief process, are helping to better clarify that complex process (Castillo et al., 2015; Connors and Halligan, 2015; Angel et al., 2017). What we need now is to better clarify the fields in which beliefs and believing play an essential role, not only a provisional one that could be replaced in short order by more reliable cognitive means.

Before going into the proposed analysis, a thesis can be proposed: beliefs are required as conditions for the formation of every social system, not just religion. The thesis can appear too bold for many, but for others, this is just a truism: without shared beliefs, we cannot conceive how systems like the economy, politics, and the judiciary could work. Some examples will suffice.

## Revisiting social systems as believing systems

Science is the first case to consider. Scientists need to hold general beliefs about the world we inhabit, its knowability, and the ability of our theories and models to represent it. Then, when scientists formulate their models based on the available data and analysis, they need to believe that those assumed will work better than alternative ones, something which cannot be taken for granted. This often opens new challenges with data and analysis that could disprove earlier models that most colleagues believed. Pluralism of methods–even in statistical analysis–requires that a researcher puts their faith in one procedure rather than another since choices are unavoidable and so do biases and assumptions. In that sense, a fallibilist model of science, as is the one inspired by Popper, cannot avoid relying on beliefs, more than on certainties. However, it is disputed to what extent scientists just “believe” or rather “know”; obviously in many cases, they know beyond doubt, while in many others their certainty levels come close to believing in the way it has been previously defined.

In the economy, things are more complex, since there are many factors involved in that human activity, and social interaction renders it less predictable. The many crises we have lived through have been not just economic or financial crises, but crises of economic models. Pluralism is present and subtle in economic theory and analysis. In such a panorama, economics as an academic activity depends to a considerable extent on shared basic beliefs and values. The issue becomes still more acute when we deal with real economic subjects: the beliefs and values that they held to determine the course of economic activity; their expectations affect decisions and behaviors. Economic functioning requires trust in other people and institutions, and this is basically a form of belief.

The economic-inspired awareness of the importance of beliefs finds a special application in a related field that now assumes an autonomous status: sustainability studies. In this case, beliefs are clearly involved in any attempt to design sustainable systems applying the standard 3 ESG dimensions: environmental, social, and governance. We can speak about a human factor deeply ingrained in programs aimed at ensuring a better future for all, or just at the endurance of social bodies. Held beliefs and values are indeed informing the behavior of producers, politicians, and consumers, and those general views will determine whether it is worthwhile to undertake some sacrifices or to pay more attention to measures targeted at saving energy and other resources.

Moving to a different area, beliefs become central in psychotherapy. Indeed, it is broadly assumed that psychological distress and suffering are often linked to wrong beliefs and that some beliefs help to cope with harsh crises, while others usually worsen personal conditions, life quality, and relationships. The point is still more evident when dealing with vaccination campaigns: believing in its efficacy contributed to preventing attitudes of resistance. Moreover, believing the goodness of treatment clearly helps its efficacy. Once more, the human factor needs to be considered in therapeutic processes, besides the usual technical issues and their effects.

Other social systems can be reviewed under those critical lenses revealing them as sets of shared beliefs. This principle applies, for instance, to the judiciary, to the political system that undergoes democracy, to education in all its stages, to the system of media and information, and even to the system of broad social interaction. We need to keep some levels of trust or belief about the reliability of those we meet and those with whom we have exchanged. Trust appears–even in the usual definitions–as a very close concept to “belief,” but clearly applied to persons: believing in somebody means trusting him or her. Up to a point we can claim that every exercise of communication involves believing that our message will reach the recipient correctly and that it will not be misunderstood, or confidence based on some “charity principle” that other persons will not be trying to fool me all the time, confidence that is not present in people afflicted with paranoid beliefs. Of course, that requirement, which is basic in everyday communication, becomes much more stringent when the interactions move toward a greater intensity, as in family, friendship, and close-knit groups.

## Discussion: Beliefs and normativity

The final points direct us toward a very sensitive issue: use and misuse of beliefs, and how to order them, or, rather, how to prevent abuses. This is a growing threat in a context dominated by new social media, with a huge flow of information, and where it becomes harder to assess which contents we can trust, in the midst of so much fake news. Believing becomes not merely a spontaneous activity, but a discipline that needs to be formed and to be built on a surer and more reliable ground. Such education programs would be aimed, for instance, to prevent predominant biases, like prestige and confirmation biases. A normative dimension derives from such awareness, a kind of “ethics of believing” should be assumed as a necessary chapter in the study and application of believing, a field that now receives more attention (Peels, 2016; Schmidt and Ernst, 2020).

The last application of believing is perhaps the most obvious, but not less subject to deep study and attention. Indeed, more analysis points to a convergence between religions and systems of meaning all placed under the umbrella of general belief systems, or sets of values, expectations, or faiths, able to provide meaning and purpose. These appear as a special kind of beliefs, with their own specific formation processes and characteristics, with central functions, and–again–unavoidable and not assimilated to other cognitive forms, like scientific scrutiny or sense perception.

All that has so far been discussed points to the importance of better studying and understanding the process of believing, often a pending issue in many areas, perhaps because of the dominance of reductionist models of cognition, which have neglected other forms as derived or secondary. We need good science to approach beliefs too, and to know better how they work, but not to replace them, something we could not, in any case, afford to do without a great anthropological and social cost.

## Author contributions

The author confirms being the sole contributor of this work and has approved it for publication.
